# Carvacrol attenuated haloperidol-induced Parkinson’s disease via TNF/NFκβ-NLRP3-mediated pyroptosis

**DOI:** 10.1186/s42826-025-00237-7

**Published:** 2025-02-05

**Authors:** Faisal Albaqami, Khawaja Waqas Ahmad, Fawad Ali Shah

**Affiliations:** 1 Department of Pharmacology and Toxicology, College of Pharmacy Prince Sattam bin Abdulaziz University, Al-Kharj, 11942 Saudi Arabia; 2 Riphah Institute of Pharmaceutical Sciences, Riphah International University, 7th Avenue, Sector G-7/4, Street 43, Islamabad, Pakistan

**Keywords:** Parkinson, Carvacrol, Inflammasome, Thalidomide, Oxidative stress, Haloperidol

## Abstract

**Background:**

Parkinson’s disease is a debilitating and the second most common neurodegenerative disorder with a high prevalence. Parkinson’s disease has a multifaceted etiology characterized by an altered redox state and an excessive inflammatory response. In this study, we investigated the potential neuroprotective properties of carvacrol in a haloperidol-induced Parkinson’s model. In female Sprague-Dawley rats, the animal Parkinson model was induced by intraperitoneally administering 1 mg / kg of haloperidol once daily for fifteen days. Carvacrol was administered at a dose of 25 and 50 mg / kg once daily for fifteen days before haloperidol administration. In order to further illustrate the vital role of the tumor necrosis factor (TNF-α) pathway, we administered 50 mg / kg of the TNF-α inhibitor thalidomide once daily for 15 days.

**Results:**

Our results showed that haloperidol-induced motor deficits, changed endogenous antioxidant enzymes, along with higher levels of inflammasome (NLRP3) and other inflammatory mediators. Moreover, increased levels of lipid peroxidase (LPO) indicated a significant rise in oxidative stress due to haloperidol. Moreover, carvacrol reduced these effects by preventing pyroptosis mediated by the inflammasome (NLRP3) and TNF-α. The administration of thalidomide mitigated oxidative stress and suppresses inflammatory pathways through the augmentation of the intrinsic antioxidant system. Further, co-treatment of carvacrol with thalidomide synergized the neuroprotective effect of carvacrol as demonstrated by various immunoassays and histology analyses.

**Conclusions:**

Taken together, our findings suggest that carvacrol mitigated haloperidol-induced Parkinson-like symptoms, partially through the downregulation of TNF-α and NLRP3.

## Background

Parkinson’s Disease (PD) is the second most prevalent age-related neurological disorder and has affected around 6.3 million people around the globe [1]. It is characterized by significant tremors, stiffness, and diverse postural abnormalities due to a deficiency of dopamine. The primary pathophysiology of PD is linked to the formation of Lewy bodies and the degeneration of dopaminergic neurons; however, current data suggests the possible impact of many other prognostic variables [2, 3]. Moreover, its distinctive pathological feature is characterized by the progressive degeneration of dopaminergic neurons in the substantia nigra pars compacta (SNpc), resulting in the depletion of dopaminergic input to the striatum. Although motor deficits can be managed by therapeutic interventions, concurrent medication is required for non-motor clinical symptoms, including depression, dementia, and different autonomic dysfunctions [4].

Numerous studies indicate that the nervous and immune systems function in harmony to preserve robust connectivity [3]. Moreover, neuroinflammation and inflammasomes such as NLRP3 contribute to the pathogenesis of PD [5, 6]. Furthermore, the activation of the inflammasome serves as a fundamental catalyst for pyroptosis activation [7]. NLRP3 belongs to the NLR family of cytosolic pattern recognition receptors and regulates the activation of caspase-1 by the assemblage of NLRP3 and other members of its family in response to pathogen-derived and danger-associated factors. Its activation is caused by reactive oxygen species and results in the release of proinflammatory cytokines such as IL-1β and TNF-α [8].

Tumor necrosis factor (TNF-α) is a vital cytokine, and dopaminergic neurons of the substantia nigra have profound sensitivity to TNF-𝛼 [9]. Furthermore, the substantia nigra (SN) and cerebrospinal fluid [CSF] of post-mortem Parkinson’s disease have been shown to have elevated TNF-α than the healthy adult brain [10]. The significance of TNF-α-mediated signaling in individuals predisposed to PD has been substantiated by animal studies that have established a correlation between elevated TNF-α levels and the onset of clinical symptoms [11]. In recent decades, thalidomide (TLD) has been repurposed for the treatment of a variety of inflammatory and cancer-related conditions. It is a TNF-α inhibitor and possesses antiangiogenic and immunomodulatory properties. The transcription of inflammatory molecules and NLRP3 inflammasome components is regulated by TNF-α, and several studies demonstrate that TNFR may be a trigger for NLRP3 [12]. Moreover, the levels of NLRP3 and TNF-α are synchronously elevated in several kinds of neurodegenerative disorders [13].

Carvacrol (CA) is a phenolic monoterpene, used as a food supplement. Previously, carvacrol showed protective effects in the experimental PD [14]. The antioxidant and anti-inflammatory properties of CA are probably responsible for its positive biological benefits. In previous studies, CA demonstrated neuroprotective effects in ischemic brain injury, and it mitigated behavioral deficits and memory impairment [15, 16]. Another study demonstrated that it can alleviate depression-like symptoms [17]. The purpose of this study is to investigate CA for its anti-pyroptosis effects in combination with TLD in the haloperidol (HP)-induced model. HP is a typical antipsychotic and can provoke extrapyramidal effects such as postural rigidity, slowness in movement (bradykinesia), and tremors epitomized in animals as catalepsy. It is frequently employed as an animal model for investigating motor impairments observed in PD and for screening potential antiparkinsonian compounds, as it triggers the blockade of D2 dopaminergic receptors in the nigrostriatal pathway. The precise mechanism by which HP mediated these effects is unknown; however, the HP metabolite pyridinium may be toxic to dopaminergic neurons [18]. Further research demonstrated that these effects of HP might be attributed to its enhanced contribution to dopamine metabolism and free radical production [19]. Further HP accelerates oxidative metabolism and caspase reactivity to produce inflammatory and oxidative pathways activation in the brain [20].

## Methods

### Chemicals and reagents

p-JNK (SC-6254), p-NFκB (SC-271908), and TNF-a (SC-52B83) were purchased from Santa Cruz Biotechnology (SCBT, USA). Elisa’s kit of rat NLRP3 (Catalog No. A5652) and TNF-α Elisa’s kit (E-EL-R0019) were purchased from Shanghai MLBIO Biotechnology Co., Ltd. (Shanghai, China), and Elabscience (Houston, TX, USA), respectively. Carvacrol (Catalog No. W224511) was purchased from Sigma (USA). All other chemicals, such as TLD and HP, were obtained from the local pharmaceutical industry with the highest analytical grade.

### Animal grouping and drug treatment

**Fig. 1:**
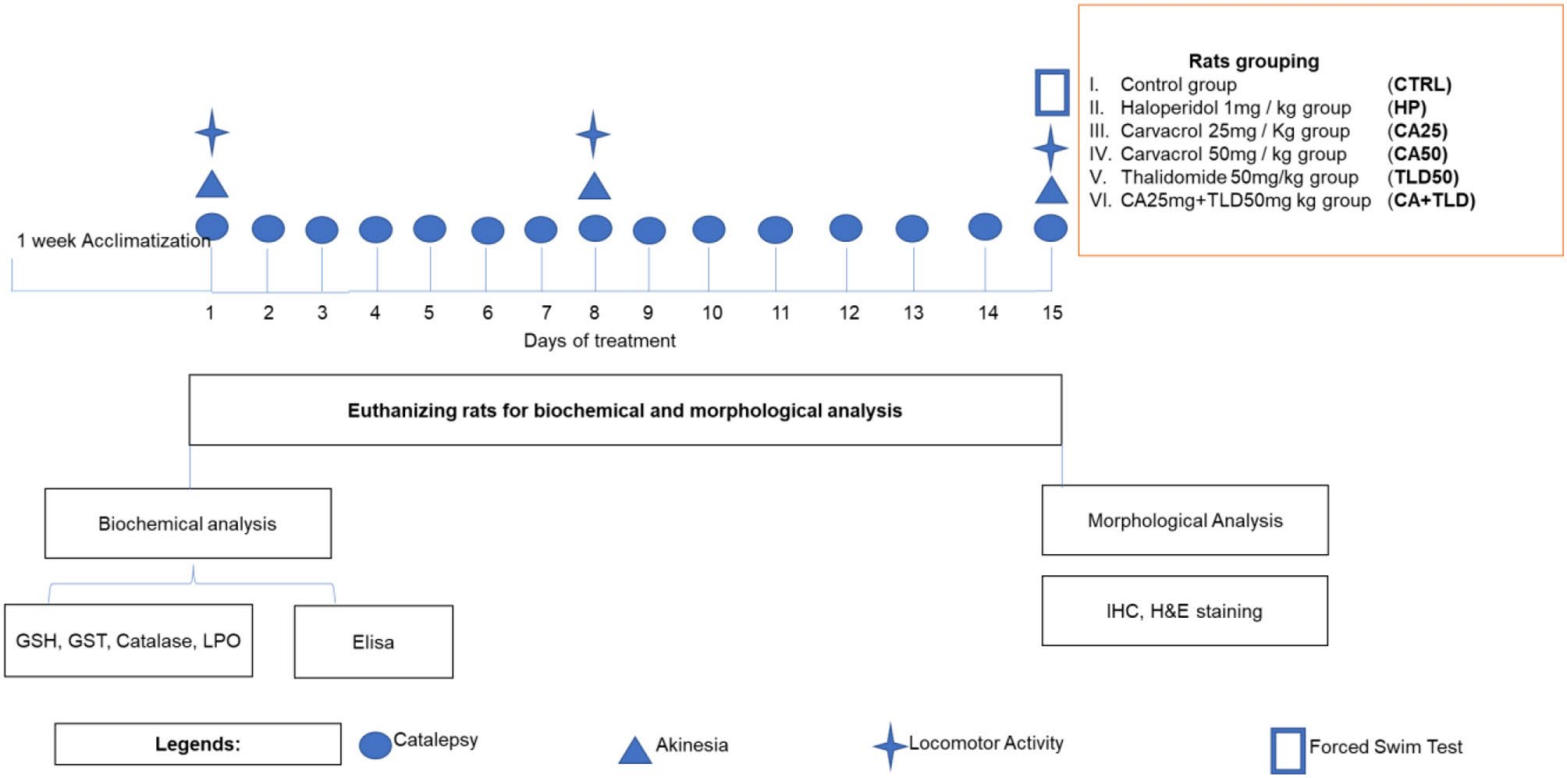
Schematic presentation of experimental design: Animals were acclimatized for one week before experimental procedures. Rats were subjected to a 15-day treatment protocol, followed by neurobehavioral analysis. Following experimentation, animals were sacrificed, and the brain was extracted and processed either for biochemical analysis and/or morphological analysis

Adult Sprague-Dawley female rats with an average weight of 230 ± 20 gm were acquired locally from the breeding facility of Riphah International University, Islamabad. All animals were housed at the Laboratory Animal Research Centre, Riphah International University, under a 12 h light/12 h dark cycle at 18–22 °C, and food and tap water access was ad libitum during the project. All experimental procedures were conducted as per protocol approved by the Institutional Animal Care and Committee of Riphah Institute of Pharmaceutical Sciences (Ref no. REC/RIPS/2020/007). All rats were acclimatized for a week before in vivo studies. Rats were divided randomly into six groups (Fig. 1). Control group (CTRL), rats in this group received normal saline once daily for fifteen days. Haloperidol (HP) group, HP was administered at a dose of 1 mg / kg once daily for fifteen days to induce PD symptoms. Carvacrol (CA25 and CA50) groups, CA was administered at a dose of 25 mg / kg and 50 mg / kg once daily for fifteen days before HP administration. Thalidomide 50 mg/kg group (TLD50), TLD was administered at a dose of 50 mg / kg once daily for fifteen days before HP administration. Carvacrol + Thalidomide + Haloperidol group (CA + TLD), pretreated with CA at a dose of 25 mg / kg followed by TLD at 50 mg / kg. All doses were administered intraperitoneally for 15 days, and a 30-minute interval was practiced between each successive dose. The selection of these doses was based on a previously used different experimental models [21–23]. After scheduled dose regimens, behavioral studies were conducted, and finally, rats were euthanized with an I/P administration of a cocktail of xylazine (9 mg / kg) and ketamine (91 mg/ kg), and brain samples were collected and divided into two cohorts. One cohort (*n* = 8/group) was processed for morphological analysis and kept at 4% paraformaldehyde solution at 2–8 °C, and the other cohort was preserved at -80 °C for biochemical analysis.

A total of 9 rats died during the experiment protocol: 1 rat from the CA50 group, 2 from TLD50, and 6 from CA + TLD on the 7th, 8th, and 10th days and were further adjusted by adding more rats. The apparent reason for these deaths in TLD administered groups was possibly that TLD might compromise the animals’ immune systems, rendering them susceptible to unexpected risks, while the possible cause of death in the CA50 group was unknown. Moreover, CA + TLD rats exhibited signs of weakness, such as eye redness and other abnormalities, along with reduced mobility and notable signs of distress.

### Haloperidol-induced catalepsy

Catalepsy was performed to record latency in removing forearms or moving the head in an exploratory manner from the externally imposed atypical posture. The forepaws of the individual rat were placed on a horizontal bar elevated 9 cm above the table surface; time was logged till the rat kept it steady on the bar or moved his head in an exploratory way with a cut-off time of 180 s. Three consecutive readings were taken every day for fifteen days.

### Haloperidol-induced akinesia

Each animal was individually placed on a wooden platform (dimensions 40 × 40 cm, 30 cm elevated above ground) for mapping latency to initiate movement, i.e., time taken by the animal in moving all four limbs. Each animal was acclimatized individually for 5 min on the said wooden platform before recording the akinesia score, and the cut-off time was 5 min and was performed at an interval of 2, 4, 6, and 8 h after drug administration on days 1, 8, and 15.

### Locomotor activity (LMA)

Rats were placed in a clean, identical cage but devoid of bedding and litter. The cage was divided virtually into four equal quadrants. Locomotor activity was measured by counting the number of lines crossing by each animal for 5 min following 4 h of dose administration and was performed on days 1, 8, and 15. The cages were cleaned with 70% ethanol in between experiments to avoid confronting factors.

### Swim test

In this test, the rat’s immobility duration was recorded by placing it in the water tank (20 × 20 × 40 cm) and maintaining 25 °C. On the 14th day, animals were trained for 15 min, and on the 15th day, animals were subjected to a forced swimming test for 5 min to record immobility time (time required to attain lack of motion of a whole body with only essential movements to retain the animal’s head above the water).

### Hematoxylin Eosin (H & E) staining

Paraffin blocks were first prepared from coronal brain slices and were later trimmed into 4 μm thin sections and fixed on slides. The tissue slides were dipped in absolute xylene (100%) for de-paraffinizing and rehydrated with graded concentrations of ethyl alcohol (from 100 to 70%). The slides underwent a series of procedures, as follows: they were immersed in hematoxylin for 10 min and subsequently submerged in running water within a glass container for an additional 10 min. Following this, they were subjected to a co-treatment involving 1% hydrochloric acid and 1% ammonia, a technique previously described [24]. After this treatment, the slides were exposed to an eosin solution for 5–10 min. Afterward, they were thoroughly rinsed with water and allowed to air-dry. The dried slides were then sequentially immersed in ethyl alcohol solutions with concentrations of 70%, 95%, and 100%. Finally, they underwent a clearing process with xylene and were mounted with a glass coverslip. To assess the slides, they were photographed employing a light microscope from Olympus, Japan, and subjected to analysis using ImageJ [25].

### Immunohistochemical analysis

Tissue blocks were sectioned into 4-micrometer-coronal slices (Leica microtome, Germony). After conducting antigen retrieval and neutralizing peroxidase activity, a 5% serum was applied. Following this, a sequence of steps was performed, commencing with the use of primary antibodies (p-JNK, p-NFκB, and TNF-α (dilution 1:100, Santa Cruz Biotechnology SCBT, USA), followed by secondary antibodies, ABC treatment (Santa Cruz Biotechnology SCBT, USA), and concluding with DAB staining. Five images per section (tissue) were captured from each group under the same conditions using a microscope (Olympus Japan). Images were saved in a tagged image file format (TIF) and converted to an 8-bit image type. The integrated density was quantified in the same region of the brain areas as the TIF images for all groups using ImageJ software. The background of TIF images was optimized to the threshold intensity, false regions were removed, and the integrated density was analyzed for p-JNK, TNF-α, and p-NFKB-positive cells at specified threshold intensity at least three times for all groups under the same conditions. The integrated intensity is expressed as the relative integrated density of the samples, and the corresponding histograms and statistical tests were done using Prism software. The bar graphs were produced from the raw integrated density of immunoreactivity in ImageJ, but for easy readership, we divided all the raw values (arbitrary unit numbers) by their average numbers, which gave us the relative integrated density values in a few numbers instead of thousands and millions of values.

### Oxidative enzyme analysis

The buffer system methodology was implied to get the levels of oxidative stress markers. The concentration of reduced glutathione (GSH) was determined by combining the supernatant with DTNB and PBS and determining the absorbance at 412 nm [26]. For GST determination, the supernatant was combined with glutathione solution and CDNB, and the absorbance was at 340 nm. For catalase activity, protein supernatant was combined with H2O2 and PBS, and absorbance was measured at 240 nm.

### LPO assay

Reactive intermediates produced by oxidative stress may alter bilayers in cell membranes, leading to polyunsaturated fatty acid (PUFA) peroxidation. This leads to the formation of lipoperoxyl radicals (LOO•), which interact with lipids to yield a lipid hydroperoxide (LOOH) and a lipid radical [27]. The lipid peroxidation (LPO) analysis was conducted following a previously described method with slight modifications [28]. The cortex and hippocampus regions were carefully isolated and separately homogenized in 10 mL of 20 mM Tris-HCl buffer (pH 7.4) at 4 °C, utilizing a polytron homogenizer. The resulting homogenate was subsequently subjected to centrifugation at 1,000 g for 10 min at 4 °C, leading to the separation of the supernatant. This supernatant was then combined with freshly prepared ferric ammonium sulfate (40 µL) and incubated at 37 °C for 30 min. Finally, 75 µL of thiobarbituric acid (TBA) was added, and the absorbance was immediately recorded at 532 nm using a microplate reader. The recorded values were expressed as Tbars-nM/min/mg protein.

### Enzyme-linked immunosorbent assay (ELISA)

Using an ELISA microplate reader, we quantified NLRP3 and TNF-α using procedures described in prior research and following the manufacturer’s guidelines.

### Statistical analysis

The data were demonstrated as means and standard errors of the means (SEM). One-way and two-way ANOVA tests, followed by Tukey’s multiple comparisons test, were applied to perform the statistical analysis; this was achieved using GraphPad Prism 5. Significant differences were specified by the symbols * and #. The symbols # is representative of significant differences between control and HP, while * between treatment groups and HP. Catalepsy and akinesia were analyzed using two-way ANOVA, whereas the swim test was by one-way ANOVA.

## Results

### Effects of carvacrol on haloperidol-induced movement impairment

**Fig. 2:**
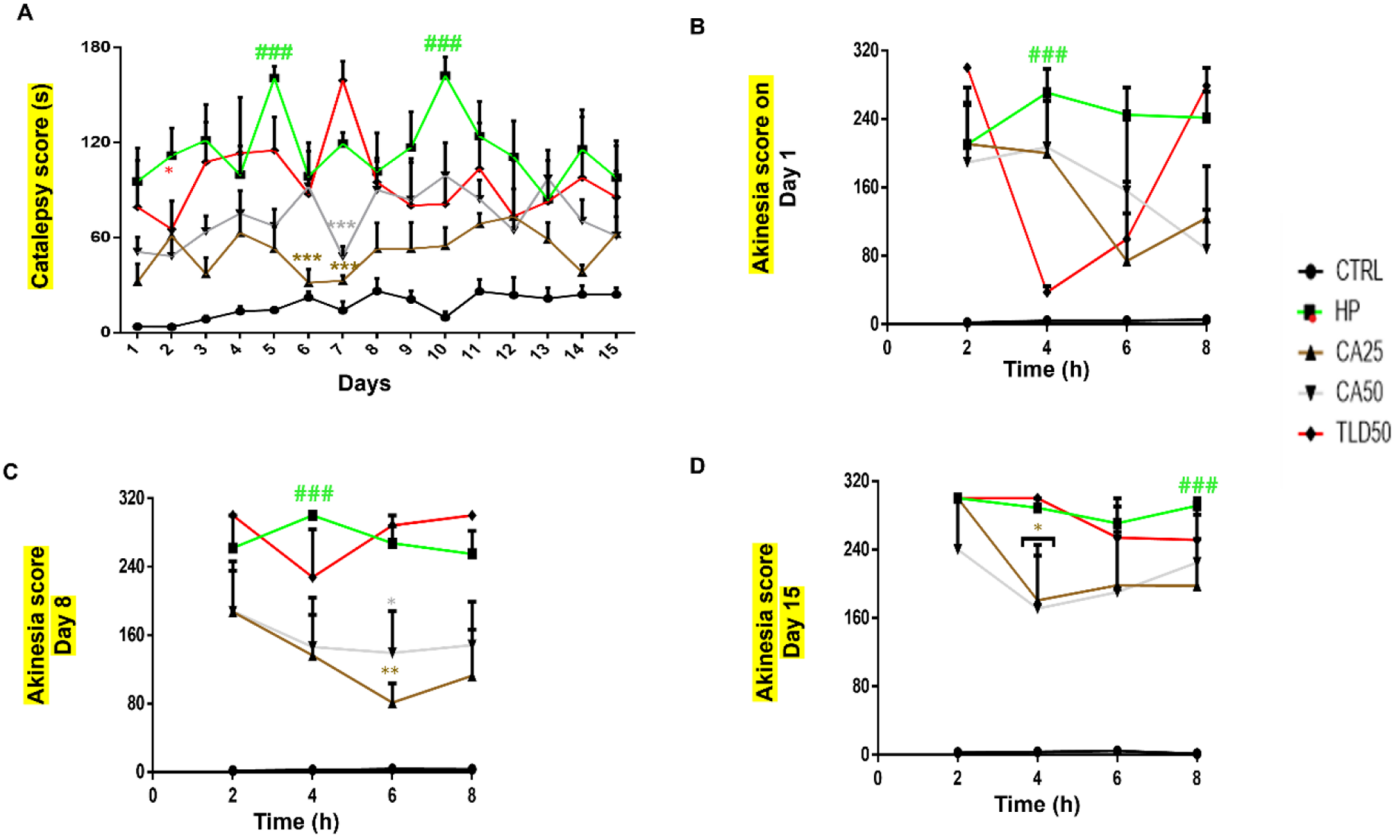
Carvacrol attenuated haloperidol-induced impaired movements. (**A**) Catalepsy score (**B**) Akinesia Day 1 (**C**) Akinesia Day 8 (**D**) Akinesia Day 15. All data is presented as mean + SEM and analyzed by two-way ANOVA followed by post hoc Tukey’s multiple comparison test

HP induced a full-blown catalepsy within an hour of its administration, as suggested by previous studies. The catalepsy score in the HP group was 114.72 s *±* 47.53 compared to the average 17.05 s *±* 13.55 in the CTRL group (Fig. 2A, ### *P* < 0.001), with a peak cataleptic score of 162.25 s noted in the HP group. The CA25 group produced a noticeable decrement in the cataleptic state, averaging 51.5 s *±* 21.75 (Fig. 2A *** *P* < 0.001) and the peak effect being 73.25 s. The CA50 group also showed visible improvement with a mean score of 73.08 s *±* 26.17 (Fig. 2A, *** *P* < 0.001) and a peak value of 99.25 s. TLD50 group averaging 95.12 s *±* 64.13 (Fig. 2A, * *P* < 0.05) with a maximum score of 159.25 s.

Only the HP group showed 270.75 s *±* 83.75 on Day 1 (Fig. 2B, ### *P* < 0.001), and the rest of the treatment groups showed insignificant changes. On day 8, the HP group’s average akinetic score was 300 s (Fig. 2C, ### *P* < 0.001), and the CA25 group showed significant progress in alleviating the akinetic state, averaging 81.25 s *±* 60.75 (Fig. 2C, ** *P* < 0.01), and the CA50 group also shown progress with an average state of 139.25 s *±* 125.25 (Fig. 2C, * *P* < 0.05). On day 15, the HP group produced akinesia for 300 s (Fig. 2D, ### *P* < 0.001) and CA25 displayed an improved average of 180.75 s *±* 132.75 (Fig. 2D, * *P* < 0.05) and CA50 attenuation of akinesia to an average of 171 s *±* 138 (Fig. 2D, * *P* < 0.05).

### Effects of carvacrol on locomotor activity

**Fig. 3:**
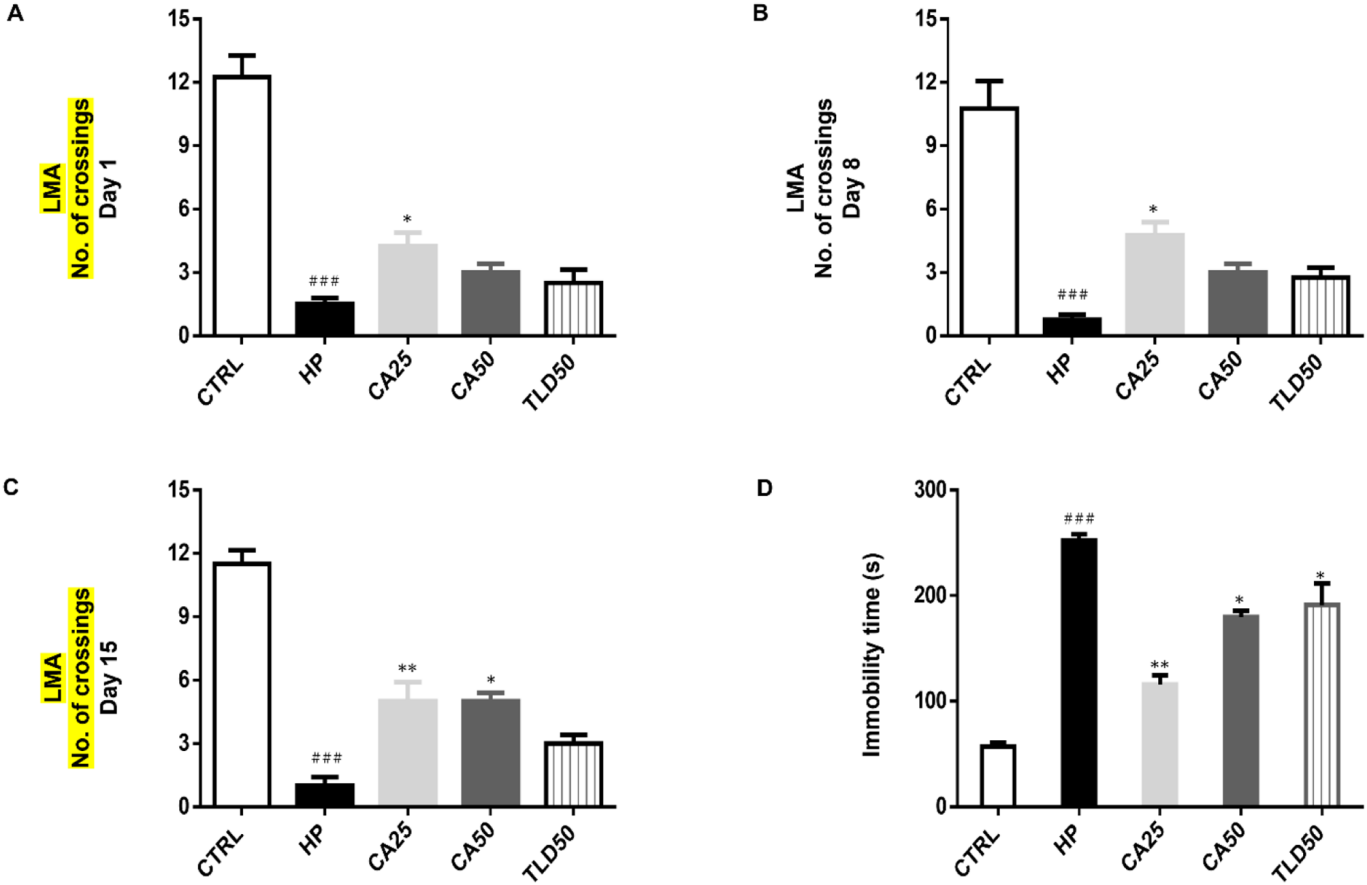
Effects of carvacrol on locomotor activity (LMA) and swim test. (**A**) LMA day 1 (**B**) LMA day 8 (**C**) LMA day 15 (**D**) A swim test was presented. All data is presented as mean ± SEM analyzed by one-way ANOVA followed by post hoc Tukey’s multiple comparison test. Symbols ### *P* < 0.001, ## *P* < 0.01, and # *P* < 0.05 represent significant differences between the CTRL group relative to the HP group, whereas *** *P* < 0.001, ** *P* < 0.01, and * *P* < 0.05 represent significant differences between the HP group compared with other treatment groups

On day one, the HP group expressed hypolocomotion with an average score of 1.5 *±* 0.5 (Fig. 3A, ### *P* < 0.001), and the CA25 group showed improved locomotion with an average score of 4.25 *±* 1.75 (Fig. 3A, * *P* < 0.05). On the 8th day, the HP group showed 0.75 *±* 0.75 (Fig. 3B, ### *P* < 0.001) while the CA25 group averaged 4.75 *±* 1.75 (Fig. 3B, * *P* < 0.05). On the 15th day, the HP group resulted in 1.0 *±* 1.0 (Fig. 3C, ### *P* < 0.001), whereas the CA25 and CA50 groups showed significant improvement with mean values of 5 *±* 2 and 5 *±* 1.0, respectively (Fig. 3C, ** *P* < 0.01 and * *P* < 0.05).

The average immobility span for the CTRL group was 57 s *±* 8, whereas for the HP group it was 252 s *±* 19 (Fig. 3D, ### *P* < 0.001), and the CA25 group exhibited significant improvement at 115.75 s *±* 22 (Fig. 3D, ** *P* < 0.01). CA50 group and TLD50 group also showed improvement of 179.75 s *±* 15.25 and 191 s *±* 65 (Fig. 3D, * *P* < 0.05).

### Carvacrol modulated NLRP3-mediated pyroptosis

**Fig. 4:**
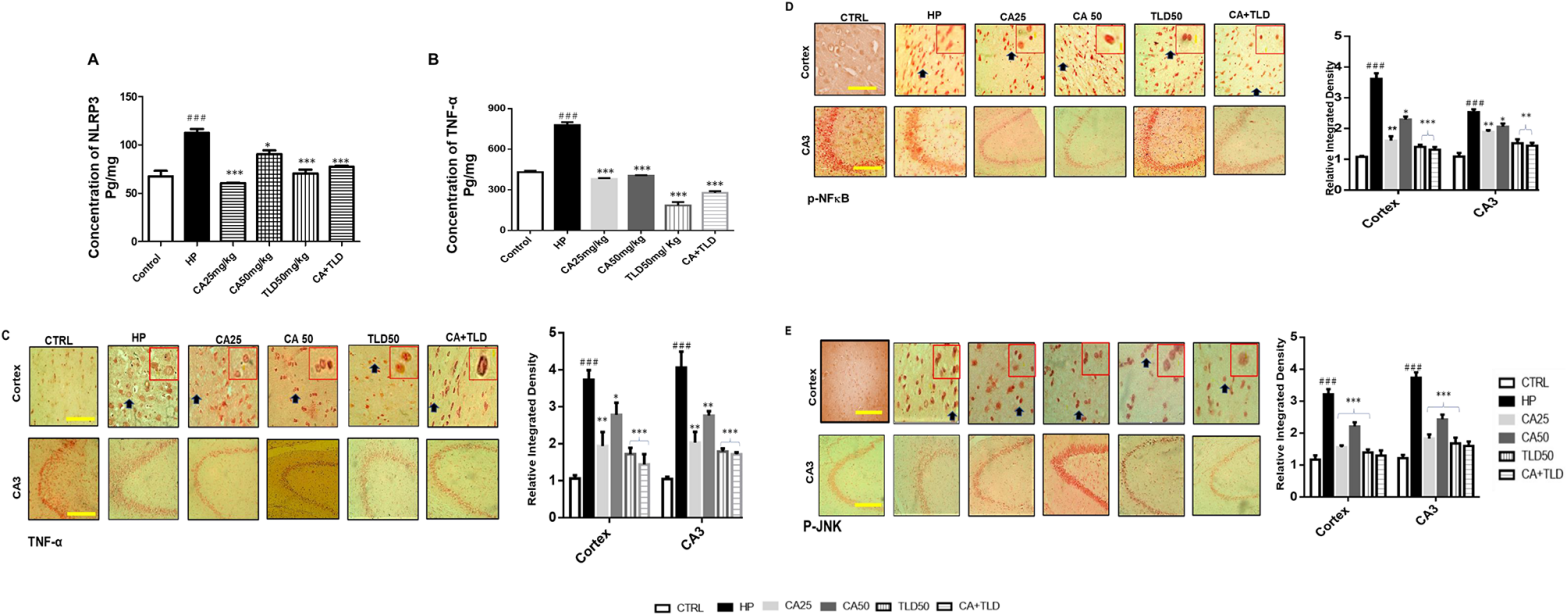
Effects of carvacrol on neuronal inflammation. (**A**) Quantification of TNF-α and NLRP3 expression in the cortical region presented as mean ± SEM by Elisa (**B**) TNF-α immunohistochemistry in cortical and hippocampal CA3 and DG regions presented as mean ± SEM. (**C**) p-NFKB immunohistochemistry in cortical and hippocampal CA3 and DG regions. (**D**) p-JNK immunohistochemistry in cortical and hippocampal CA3 regions. All data was analyzed by a two-way ANOVA followed by a post hoc Tukey’s multiple comparison test. Symbols ### *P* < 0.001, ## *P* < 0.01, and # *P* < 0.05 represent significant differences relative to the HP group, whereas *** *P* < 0.001, ** *P* < 0.01, and * *P* < 0.05 represent significant differences relative to the treatment groups

The NLRP3 inflammasome-induced inflammatory cascades are significantly dependent on the activation of the inflammatory pathway. To further examine, protein levels of NLRP3 and TNF-α were determined by Elisa. Treatment with CA50 and TLD50 significantly reduced the protein levels of NLRP3 (* *P* < 0.05, *** *P* < 0.001, Fig. 4A) and TNF-α in the cortex (*** *P* < 0.001, Fig. 4B), which were raised in the HP-treated group (Fig. 4A and B). However, CA25 produces a more significant effect against NLRP3 (*** *P* < 0.001, Fig. 4A) in the cortex. Similarly, the expression of other inflammatory markers was also validated by immunostaining for TNF-α, p-NFκβ, and p-JNK (Fig. 4C, D and E). The results demonstrated that the level of all these inflammatory mediators and markers was increased in the HP group (### *P* < 0.001). Moreover, it was observed that the effect of CA25 was more substantial than that of CA50 for TNF-α and p-NFκβ, whereas the differences were comparable for p-JNK (Fig. 4C and D).

### Effects of carvacrol on neuronal insult

**Fig. 5:**
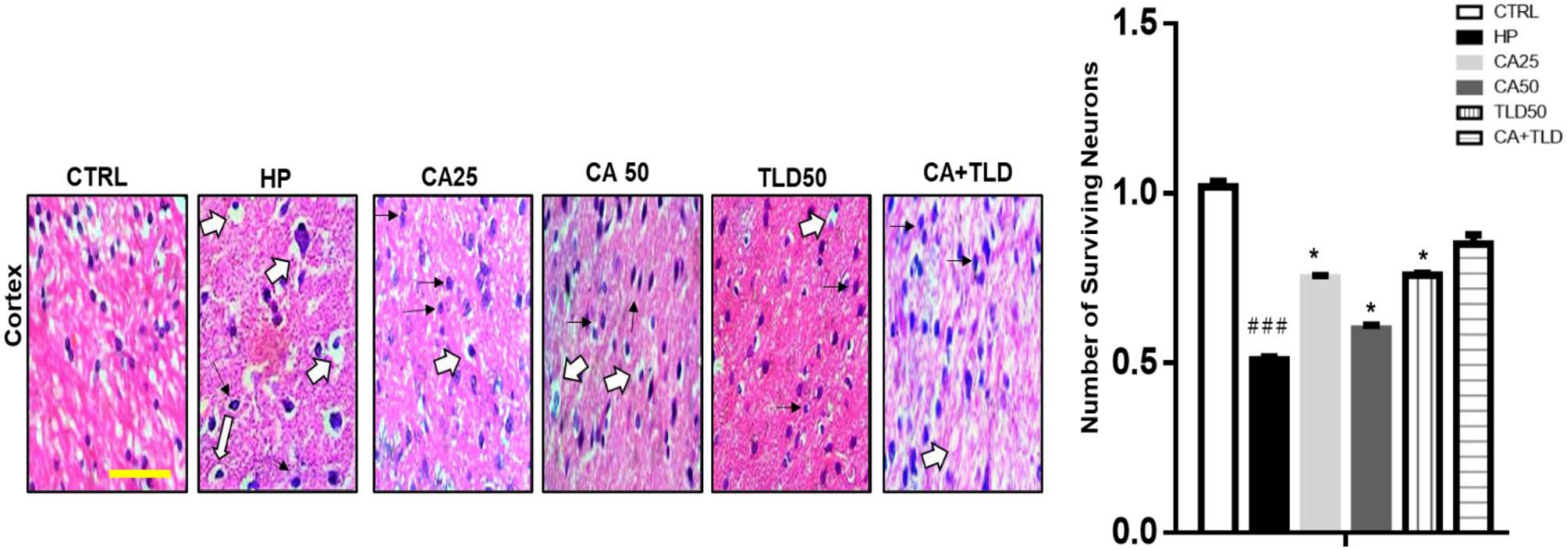
Carvacrol attenuated HP-induced neurodegeneration. H&E staining shows the extent of surviving neurons in the cortex. Scale bar 50 μm, magnification 40×. Deranged neurons were characterized by swollen cytoplasm, vacuolization, scalloped morphology with intense cytoplasmic eosinophilia, and nuclear basophilia. Data are expressed as means ± SEM. ### *P* < 0.001 compared to the control group, while ** *P* < 0.01, *** *P* < 0.001 compared to the HP. CAR 20: carvacrol (20 mg / kg); CAR 50: carvacrol 50 mg / kg

Deranged neurons were characterized by swollen cytoplasm, vacuolization, scalloped morphology with intense cytoplasmic eosinophilia, and nuclear basophilia. Relative injury in the cortex region was determined by assessing vacuole formation and damaged and scalloped-shaped neurons, and the extent of these neurons was found more in the HP group (### *P* < 0.001, Fig. 5), which was ameliorated by CA25, CA50, and TLD50 comparably (* *P* < 0.05, Fig. 5).

### Carvacrol upregulated endogenous antioxidative enzymes

**Fig. 6:**
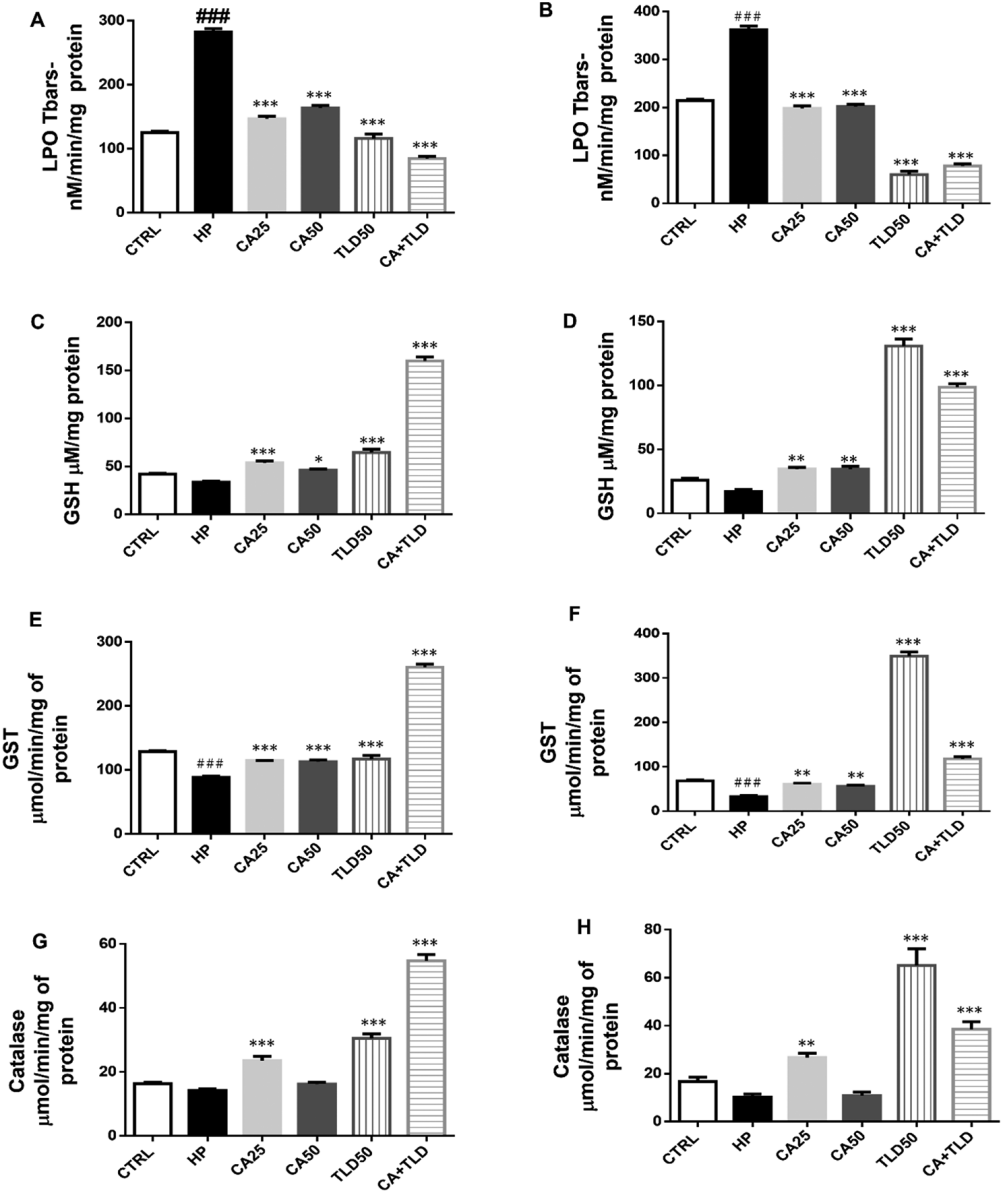
Carvacrol attenuated antioxidant enzyme levels. (**A**) LPO level in the cortex (**B**) LPO level in the hippocampus (**C**) GSH in the cortex (**D**) and GSH in the hippocampus (**E**) GST in the cortex (**F**) GST in the hippocampus (**G**) Catalase in the cortex (**H**) Catalase in the hippocampus. All data were presented as means ± SEM and analyzed by one-ANOVA followed by post hoc Tukey’s multiple comparison test. Symbols ### *P* < 0.001, ## *P* < 0.01, and # *P* < 0.05 represent significant differences relative to the HP group, whereas *** *P* < 0.001, ** *P* < 0.01, and * *P* < 0.05 represent significant differences between the treatment groups

Inflammation and oxidative stress are prominent characteristics of neurodegenerative disorders. The TBARS technique was often used to evaluate the lipid peroxidation of polyunsaturated fatty acids, which produces reactive aldehydes. Our TBARS findings demonstrated an upsurge in LPO level (Fig. 6A) in the HP group (282.53 ± 9.6) compared to control (125.05 ± 4.38) and treatment groups significantly (*** *P* < 0.001) reversed LPO level comparable to HP group for CA25 (146.77 ± 7.48), CA50 (163.99 ± 7.13), TLD50 (116.43 ± 7.27) and CA + TLD (84.75 ± 6.92). Correspondingly, hippocampal homogenates exhibited a rise in LPO level in HP (362.24 ± 13.92), and treatment groups showed a statistically significant reversal in LPO content (*** *P* < 0.001, Fig. 6B).

Brain antioxidant capacity was also evaluated by measuring antioxidant enzymes GST, catalase, and glutathione reduced form. GSH (Fig. 6C and D), GST (Fig. 6E and F), and catalase (6G, 6 H) levels were significantly reduced in both cortical and hippocampal homogenate. However, treatment with carvacrol and thalidomide significantly restored the level of these antioxidant enzymes.

## Discussion

Extensive research has been conducted on natural compounds to identify potential treatments and experimental methodologies for PD, and a diverse range of natural compounds that have proven efficacious in PD models, both in vivo and in vitro, have been identified. These substances target several important areas linked to the pathophysiology of PD [14]. The present study focuses on CA to assess its potential neuroprotective and anti-inflammatory properties in HP-induced PD. We demonstrated here that CA potentially reduced inflammation by immunomodulating TNF-α and NRLP3, mitigating oxidative damage, and reversing neuronal damage, thereby ameliorating the motor deficits induced by HP. Furthermore, we examined the effects of the proinflammatory cytokine TNF-α by employing its inhibitor, TLD, both alone and in conjunction with CA. Our findings suggested that the administration of TLD not only improved motor impairments but also enhanced the effects of CA via modulating the expressions of oxidative enzymes and cytokines.

Individuals experiencing symptoms of PD are very susceptible to developing dementia, as dementia prevalence in PD patients varies from 10 to 30%, making it the second most frequent cause of dementia after Alzheimer’s disease (AD). We have used hippocampal tissue here because hippocampal atrophy consistently is associated with PD [29]. Comparable results are seen in dementia with Lewy bodies (DLB), where diffuse Lewy bodies and the clinical characteristics of AD co-occur frequently [30]. Furthermore, distinct aberrant characteristics are shown by hippocampus neurons in PD related to depression and fatigue [31]. Similarly, cognitive abnormalities in PD also include the hippocampal cholinergic and noradrenergic systems [32].

While the slowness of movement linked to PD is replicated by neurobehavioral tests like catalepsy and akinesia [33], whereas both LMA and swim tests indicate that overall motility is typically impaired with PD [34]. In PD, a decrease in dopamine levels within the nigrostriatal system is associated with neurobehavioral changes and impaired brain functions. Consequently, catalepsy is quite an effective modality for investigating neurobehavioral as well as neurochemical alterations like in PD [33]. It is widely recognized that dopamine receptor antagonists can induce cataplexy, which implies that dopamine receptor agonists may be able to alleviate these motor deficits and supports the modulatory function of dopamine receptors [33]. Our CA and TLD-treated groups expressed reversion of akinetic and cataleptic states comparable to the HP group. Likewise, the swim test demonstrated a reduction in immobility duration when CA and TLD were administered, leading to an enhancement, and an overall improvement in locomotor activity was observed as a result of this treatment.

Neuroinflammation has been extensively probed as a pathogenic component in neurodegenerative disorders, with substantial data establishing a linkage between it and the exacerbation of symptoms through ROS-induced oxidative stress. Moreover, the overproduction of free radicals is a pivotal factor in the activation of NFκB transcription factors and TNF-α [35, 36]. Our findings illustrated that the rise in LPO levels corresponded with increased NFκB and TNF-α levels, signifying the existence of cellular damage mediated by free radicals. Furthermore, inhibiting NFκB activity resulted in a reduction of its downstream inflammatory mediators, such as COX2 and iNOS, in several consistent studies [35]. Moreover, there is a cross-linkage between NFκB and TNF-α via TNFR-associated factor 1 (TRAF1) and cellular inhibitor of apoptosis proteins (cIAP1) [9].

In the present investigation, we have observed a notable upsurge in the inflammasome, which further contributes to pyroptosis. Similar to other types of cell death, pyroptosis is initiated by oxidative stress triggered by perturbations to the cellular redox balance [37]. The results of our study demonstrated a strong association between the increased frequency of pyroptosis and the elevated levels of LPO. This suggests that oxidative stress plays a pivotal role in driving pyroptosis during the development of PD. Further studies have established a correlation between elevated LPO levels and neutrophil infiltration [38]. In our study, CA and TLD treatment led to a decrease in LPO levels, which in turn correlated with a reduction in indirect neutrophil infiltration. This reduction in neutrophil infiltration subsequently inhibited the provocation of inflammation by down-regulating TNF-α and NFκB. Furthermore, the NLRP3 inflammasome plays a critical role in the development of neurodegeneration in Parkinson’s disease, as multiple findings relate the activation of the NLRP3 inflammasome to the advancement of dopaminergic neurodegeneration and the onset of motor symptoms [39]. Hence, targeting the NLRP3 inflammasome emerges as a promising approach for the development of anti-PD medications [40].

TNF-α is known to activate microglia in the midbrain, triggering inflammatory processes that lead to increased formation of reactive oxygen species (ROS), therefore worsening dopamine neuronal damage [9]. Our findings indicate reduced levels of lipid peroxidation in the CA and TLD groups. Necrotic cell death of neurons may also result from TNF-α-induced ROS generation. However, the effects of CA and TLD on H&E staining show that a higher percentage of viable neurons survive, supporting the neuroprotective role of CA. Consistent studies have underscored the protective and anti-inflammatory effects of CA and has been shown to decrease TNF-α levels in the hippocampus and mitigate LPS-induced memory impairment [41]. In another study, CA alleviated mechanical hypernociception and suppressed the inflammatory response by downregulating TNF-α expression [42]. Recent research has also revealed that CA and plant extracts rich in CA alleviate the suppression of the NF-κB signaling pathway, consequently downregulating the expression of pro-inflammatory TNF-α in diverse inflammatory conditions [43] and in cancer cell lines [44].

Symptoms of weakness have been observed in the CA + TLD group, and the precise cause is unknown. However, it’s plausible that drug interactions could occur, leading to unforeseen side effects or alterations in pharmacokinetics [45]. Additionally, TLD might compromise the animals’ immune systems, rendering them susceptible to such unexpected risks [46].

This study also presents certain limitations that we intend to address in future research. For instance, we investigated two doses of carvacrol (25 mg and 50 mg), and the lack of a dose-response analysis of CA is a limitation of the present study. Further, we did not provide detailed mechanistic insights into how CA exerts its neuroprotective effects by modulating pyroptosis. More studies are needed to elucidate the underlying mechanisms.

## Conclusions

In conclusion, our work demonstrated that CA can prevent the onset and progression of PD by downregulating the inflammasome and other inflammatory mediators, which subsequently prevents pyroptosis. These findings have relevance for possible PD treatment and diagnosis targets.

## Data Availability

The datasets used and/or analysed during the current study are available from the corresponding author on reasonable request.

## Abbreviations

CA: Carvacrol
CTRL: Control group
HP: Haloperidol
LPO: Lipid peroxidase
PD: Parkinson’s Disease
SN: Substantia nigra
TLD: Thalidomide
TNF-α: Tumor necrosis factor
